# Prevalence of Local Postoperative Complications and Breast Implant Illness in Women With Breast Implants

**DOI:** 10.1001/jamanetworkopen.2022.36519

**Published:** 2022-10-07

**Authors:** Annemiek S. Lieffering, Juliëtte E. Hommes, Lotte Ramerman, Hinne A. Rakhorst, Marc A. M. Mureau, Robert A. Verheij, René R. W. J. van der Hulst

**Affiliations:** 1 Netherlands Institute for Health Services Research (Nivel), Utrecht, the Netherlands; 2 Tranzo, Tilburg School of Social Sciences and Behavioral Research, Tilburg University, Tilburg, the Netherlands; 3 Department of Plastic, Reconstructive and Hand Surgery, Zuyderland Medical Centre, Heerlen & Sittard, the Netherlands; 4 Department of Plastic, Reconstructive and Hand Surgery, and GROW School for Oncology and Reproduction, Maastricht University Medical Center, Maastricht, the Netherlands; 5 Department of Plastic, Reconstructive and Hand Surgery, Medical Spectrum Twente, Enschede, the Netherlands; 6 Department of Plastic and Reconstructive Surgery, Erasmus MC Cancer Institute, University Medical Center Rotterdam, Rotterdam, the Netherlands

## Abstract

**Importance:**

It is unknown how often breast implant illness (BII) is the indication for revision in women with silicone breast implants.

**Objective:**

To examine how often women with silicone breast implants have their implants explanted or replaced because of BII compared with local postoperative complications.

**Design, Setting, and Participants:**

A legacy cohort study on breast implant revision surgery was conducted between April 1, 2015, and December 31, 2020, and a prospective cohort study on breast implantation and revision surgery was conducted between April 1, 2015, and December 31, 2019 (with follow-up until December 31, 2020). Data were obtained from the Dutch Breast Implant Registry. Data analysis was performed from September 2021 to August 2022.

**Exposures:**

Silicone breast implant.

**Main Outcomes and Measures:**

Breast implant revision with the indication BII or local postoperative complications.

**Results:**

All 12 882 cosmetic breast implants (6667 women; mean [SD] age, 50.6 [12.7] years) and 2945 reconstructive breast implants (2139 women, mean [SD] age, 57.9 [11.3] years) in the legacy cohort and all 47 564 cosmetic breast implants (24 120 women, mean [SD] age, 32.3 [9.7] years) and 5928 reconstructive breast implants (4688 women, mean [SD] age, 50.9 [11.5] years) in the prospective cohort were included for analysis. In the prospective cohort, 739 cosmetic breast implants (1.6%) were revised after a median (IQR) time to reoperation of 1.8 (0.9-3.1) years, and 697 reconstructive breast implants (11.8%) were revised after a median (IQR) time to reoperation of 1.1 (0.5-1.9) years. BII was registered as the reason for revision in 35 cosmetic revisions (4.7%) and 5 reconstructive revisions (0.7%) in the prospective cohort, corresponding to 0.1% of the inserted implants. In the legacy cohort, 536 cosmetic revisions (4.2%) and 80 reconstructive breast implant revisions (2.7%) were performed because of BII.

**Conclusions and Relevance:**

In this cohort study of women with silicone breast implants, BII was an uncommon indication for revision compared with local complications, both in the short and long term. In contrast to the increasing public interest in BII, these results showed that local complications are a far more common reason for breast implant revision.

## Key Points

**Question:**

How often do women have their silicone breast implants explanted or replaced because of breast implant illness (BII) compared with local postoperative complications?

**Findings:**

In this cohort study, among women who had silicone breast implants for cosmetic purposes, BII was registered as indication for revision in 4.2% of revisions in a legacy cohort and 4.7% of revisions in a prospective cohort. Among women who had silicone breast implants for reconstructive reasons, BII was registered as the revision indication in 2.7% of revisions in a legacy cohort and 0.7% of revisions in a prospective cohort.

**Meaning:**

These findings suggest that BII is an uncommon indication for revision in women with silicone breast implants.

## Teaser

This cohort study examines how often women with silicone breast implants have their implants explanted or replaced because of breast implant illness compared with local postoperative complications.

## Introduction

Breast implant surgery generally increases patient satisfaction with breasts and improves health-related quality of life.^1,2,3^ However, breast implants are also associated with long-term local complications, such as capsular contracture and breast pain.^4,5,6^ As a result, revision, rehospitalization, or other medical interventions are required over time.

In addition to local complaints, systemic health complaints in women with breast implants are receiving growing attention in the literature and especially in the lay media.^7,8^ A wide range of symptoms has been reported, including myalgia, chronic fatigue, and neurologic manifestations, which are collectively referred to as breast implant illness (BII).^9,10,11^ As public awareness of BII increases, a growing number of women are concerned about the safety of their implants in relation to BII and seek consultation with their plastic surgeon.^12,13^

Although the impact of BII is becoming more apparent, the definition of BII, as well as its existence and prevalence remain unclear.^14^ A clear need exists among patients, medical specialists, legislators, and the industry, not only for a validated diagnosis, but also for scientific evidence on the causes and prevalence of BII. Examining both local postoperative complications and BII in the same population of women could offer insight into the impact of BII compared with other complications. Therefore, the aim of this cohort study was to investigate how often women have their breast implants explanted or replaced because of BII compared with other local complications and to examine differences in patient characteristics between women with BII and women with local complications. Furthermore, this study aimed to provide insight into the revision surgery characteristics of both complication groups.

## Methods

### Privacy and Ethics

Ethics approval for this study was waived by the medical ethics committee of the University Medical Centre Maastricht. Obtaining informed consent from patients or approval by a medical ethics committee is not obligatory for observational studies using electronic health records if the database does not contain directly identifiable data (article 24 GDPR Implementation Act, article 9.2 sub[j] GDPR). This report follows the Strengthening the Reporting of Observational Studies in Epidemiology (STROBE) reporting guideline for cohort studies.

### Dutch Breast Implant Registry

The Dutch Breast Implant Registry (DBIR) is a nationwide, population-based registry. Since April 2015, DBIR has registered patient characteristics, surgical techniques, and implant characteristics for all patients undergoing breast implant surgery in the Netherlands.^15^ In contrast to other breast implant registries, DBIR uses an opt-out system, meaning that data are registered unless a patient explicitly objects. DBIR is the first breast implant registry worldwide to include the variable BII as an indication for revision surgery.

Registration in DBIR is mandatory for all plastic surgeons, who are the only medical specialists performing cosmetic and reconstructive breast implant (revision) surgery in the Netherlands. In total, 95% to 100% of hospitals and 69% to 94% of private clinics in the Netherlands performing breast implant surgery actively registered in DBIR between 2016 and 2020.^16,17,18,19^

### Study Population

For this registry-based study, 2 cohorts were selected from DBIR: a legacy breast implant cohort and a prospective cohort (eFigure 1 in the Supplement). The legacy breast implant cohort comprises patients with a record of breast implant revision surgery (replacement or explantation) performed between April 1, 2015, and December 31, 2020, without an available record of the primary breast implant insertion in DBIR. Records of implantation were missing because implantation was performed before the start of DBIR, was performed at a clinic or by a surgeon in the Netherlands not registering in DBIR, or was performed abroad (eFigure 2 in the Supplement).

The prospective cohort consists of patients with a record of primary breast implant insertion performed between April 1, 2015, and December 31, 2019, with or without a subsequent revision record. Follow-up continued until December 31, 2020, allowing for a minimum follow-up of 1 year for each patient. Primary breast implant insertion includes implantation surgery with a permanent prosthesis and staged implant-based reconstruction surgery in which a tissue expander is replaced with a permanent prosthesis.

To be eligible for either cohort, complete information on date of birth, date of surgery, indication for surgery, type of surgery, and device type (permanent implant or tissue expander) was required. Men, patients younger than 18 years, and implantation procedures in which tissue expanders were inserted were excluded.

In both cohorts, 2 groups were identified: cosmetic augmentation and breast reconstruction. The breast reconstruction group included patients who received a breast implant after mastectomy for cancer or prophylactic reasons. Patients who underwent reconstructive breast implant surgery for benign reasons or congenital deformities were excluded because of the small population size of this subcohort.

### Patient and Surgery Characteristics

Patient characteristics included age, preoperative health based on the 6-point American Society of Anesthesiologists (ASA) physical status classification, with a score of 1 indicating a healthy patient,^20^ and history of radiotherapy. Surgical characteristics were provided at breast level and included information on side of surgery and additional surgical techniques including mastopexy, flap cover, and fat grafting.

### Local Postoperative Complications and BII

Breast implant revision surgery was defined as replacement or explantation of a breast implant. DBIR collects the surgical indication, which may be single or multiple complications. Two types of complications were distinguished: local postoperative complications and BII, a systemic complication. Local postoperative complications included capsular contracture (Baker grade I to IV), asymmetry, breast pain, implant malposition, deep wound infection, skin necrosis, dissatisfaction with volume, seroma, skin scarring, flap problem, implant rupture, hematoma, silicone extravasation, and breast implant–associated anaplastic large cell lymphoma. BII was registered by the surgeon according to their judgment of nonspecific health complaints by the patient or by request of the patient. There is no objective definition of BII, which is in line with the current literature.

### Statistical Analysis

The primary outcomes in this study were the occurrence of BII and local postoperative complications as indications for breast implant revision between 2015 and 2020. Complication frequencies in the legacy cohort of revised breast implants are presented together with the median (IQR) time to reoperation for each local complication and BII. Time to reoperation of the legacy cohort was calculated from the registered implantation year of the revised implant until the year in which the implant was revised. Descriptive statistics were used to provide characteristics of patients and revision surgery in subgroups of women with BII and women with local complications. Differences in age, ASA classification, and history of radiotherapy between these 2 subgroups were tested with Fisher exact test, with *P* < .05 considered statistically significant using 2-sided tests.

For the prospective cohort, the rate of revision was calculated for the total number of breast implants. Time to reoperation of the revised breast implants was based on the date of implantation and the date of revision. Percentages of complications were calculated relative to the total number of revisions. Revision characteristics were provided separately for procedures performed because of BII and revisions because of local complications. STATA statistical software version 16.1 (StataCorp) was used for all statistical analyses. Data analysis was performed from September 2021 to August 2022.

## Results

### Legacy Breast Implant Cohort

In total, 12 882 revised cosmetic breast implants (6667 women; mean [SD] age at revision, 50.6 [12.7] years) and 2945 revised reconstructive breast implants (2139 women; mean [SD] age at revision, 57.9 [11.3] years; data not shown) were included in the legacy cohort (Table 1). The most common reason for breast implant revision in both groups was capsular contracture (4775 cosmetic revisions [37.1%] and 1115 reconstruction revisions [37.9%]), followed by implant rupture in the cosmetic group (2893 revisions [22.5%]) and breast pain in the reconstructive group (714 revisions [24.2%]). Five hundred thirty-six breast implants (4.2%; 283 women) were revised because of BII in the cosmetic group. Most revisions were performed in 2019 and 2020. The median (IQR) time between the last implant insertion and revision because of BII was 12.0 (7.0-16.0) years in the cosmetic group. In 52 of 536 breasts with BII (9.7%), the implant was ruptured. After a median (IQR) time to reoperation of 9.0 (5.0-11.0) years, 80 breast implants (2.7%; 58 women) in the reconstructive group were revised because of BII.

**Table 1.** Complications in the Legacy Breast Implant Cohort

Complications as indication for revision^a^	Cosmetic augmentation (n = 12 882)		Breast reconstruction (n = 2945)	
	Revisions, No. (%)	Time to reoperation, median (IQR), y^b^	Revisions, No. (%)	Time to reoperation, median (IQR), y^b^
Capsular contracture	4775 (37.1)	15.0 (11.0-22.0)	1115 (37.9)	10.0 (6.0-14.0)
Baker grade IV	3038 (23.6)	16.0 (11.0-23.0)	766 (26.0)	10.0 (6.0-15.0)
Implant rupture	2893 (22.5)	17.0 (12.0-25.0)	420 (14.3)	12.0 (8.0-16.0)
Dissatisfaction with volume	2176 (16.9)	12.0 (7.0-16.0)	347 (11.8)	8.0 (4.0-11.0)
Breast pain	1922 (14.9)	15.0 (10.0-20.0)	714 (24.2)	8.0 (5.0-12.0)
Asymmetry	1342 (10.4)	13.0 (8.0-18.0)	617 (21.0)	8.0 (5.0-12.0)
Silicone extravasation	1475 (11.5)	18.0 (12.0-29.0)	187 (6.4)	12.0 (8.0-17.0)
Implant malposition	860 (6.7)	12.0 (7.0-18.0)	332 (11.3)	8.0 (5.0-12.0)
Breast implant illness				
Total^c^	536 (4.2)	12.0 (7.0-16.0)	80 (2.7)	9.0 (5.0-11.0)
2015	0	NA	0	NA
2016	0	NA	1 (<0.1)	NA
2017	21 (0.2)	13.0 (10.0-19.0)	7 (0.2)	11.0 (7.0-12.0)
2018	40 (0.3)	14.0 (4.0-19.0)	14 (0.5)	5.0 (5.0-6.0)
2019	145 (1.1)	11.0 (6.0-15.0)	30 (1.0)	8.0 (5.0-11.0)
2020	330 (2.6)	12.0 (8.0-17.0)	28 (1.0)	10.0 (6.0-13.0)
With implant rupture	52 (0.4)	16.0 (11.5-19.5)	5 (0.2)	5.0 (2.0-11.0)
With silicone extravasation	44 (0.3)	15.0 (12.0-21.0)	3 (0.1)	NA
With capsular contracture Baker grade IV	58 (0.5)	15.0 (12.0-20.0)	5 (0.2)	6.0 (3.0-8.0)
With deep wound infection	9 (0.1)	14.0 (12.0-16.0)	1 (<0.1)	NA
Seroma	158 (1.2)	9.5 (6.0-15.0)	66 (2.2)	9.5 (6.0-14.0)
BIA-ALCL suspected	131 (1.0)	10.0 (7.0-15.0)	43 (1.5)	7.0 (6.0-11.0)
Deep wound infection	75 (0.6)	8.5 (3.5-15.0)	55 (1.9)	6.5 (6.0-12.0)
Skin scarring	40 (0.3)	11.0 (6.0-16.0)	63 (2.1)	6.0 (2.0-9.0)
Skin necrosis	24 (0.2)	11.0 (2.0-14.0)	26 (0.9)	8.0 (6.0-10.5)
Flap problem	15 (0.1)	8.0 (7.0-14.0)	31 (1.1)	9.0 (3.0-11.0)
Hematoma	18 (0.1)	9.0 (7.0-11.0)	10 (0.3)	NA
BIA-ALCL confirmed	12 (0.1)	10.0 (7.0-18.0)	9 (0.3)	7.0 (6.5-8.5)
Complication unknown	2930 (22.7)	14.0 (9.0-17.0)	502 (17.1)	9.0 (5.0-14.0)
Complication known for the other breast side^d^	1481 (11.5)	14.0 (9.0-18.0)	177 (6.0)	10.0 (7.0-14.0)

Abbreviations: BIA-ALCL, breast implant–associated anaplastic large cell lymphoma; NA, not applicable.

^a^ Multiple complications can be registered per breast implant; consequently, percentages do not add up to 100%.

^b^ Time to reoperation was unknown for 3759 cosmetic breast implants (29.2%) and 1281 reconstructive breast implants (43.5%).

^c^ This includes observations of breast implant illness in combination with other complications.

^d^ There is a complication registered for 1 breast and the revision is performed bilaterally.

In both the cosmetic and reconstruction group, revisions because of BII most often consisted of explantation without additional surgical techniques (265 cosmetic revisions [49.4%] and 56 reconstruction revisions [70.0%]) (Table 2). Fat grafting was a frequently used technique in addition to explantation because of BII in the cosmetic group (191 revisions [35.6%]). Revisions after local complications were most often breast implant replacements in both groups (6310 cosmetic revisions [67.0%] and 1441 reconstruction revisions [61.0%]). In the cosmetic group, women with revision surgery because of BII were younger (Fisher exact test, *P* < .001), had a lower ASA score (Fisher exact test, *P* < .001), and more often had a history of radiotherapy at revision (14 women [5.0%] vs 51 women [1.0%]; Fisher exact test, *P* < .001) compared with the group of 4993 women who underwent revision for local breast implant complications (Table 3).

**Table 2.** Characteristics of Revisions in the Legacy Cohort, by BII and Local Complications as Indication for Revision^a^

Revision surgery type	Revisions, No. (%)			
	Cosmetic augmentation		Breast reconstruction	
	BII (n = 536)	Local complication (n = 9416)	BII (n = 80)	Local complication (n = 2363)
Replacement surgery				
With implant	37 (6.9)	6310 (67.0)	9 (11.3)	1441 (61.0)
With implant and mastopexy	1 (0.2)	518 (5.5)	0	28 (1.2)
With implant and fat grafting	2 (0.4)	40 (0.4)	0	111 (4.7)
With implant and autologous flap	0	19 (0.2)	6 (7.5)	199 (8.4)
With implant and combination of techniques	0	24 (0.3)	0	19 (0.8)
Explantation				
Without additional surgical techniques	265 (49.4)	1948 (20.7)	56 (70.0)	446 (18.9)
With mastopexy	22 (4.1)	474 (5.0)	3 (3.8)	27 (1.1)
With fat grafting	191 (35.6)	62 (0.7)	3 (3.8)	16 (0.7)
With autologous flap	0	0	3 (3.8)	70 (3.0)
With a combination of techniques	18 (3.4)	21 (0.2)	0	6 (0.3)

Abbreviation: BII, breast implant illness.

^a^ Revised breasts with unknown complications are not included.

**Table 3.** Patient Characteristics of Women With BII and Women With Any Local Complication in the Legacy Breast Implant Cohort^a^

Patient characteristics	Participants, No. (%)			
	Cosmetic augmentation		Breast reconstruction	
	BII (n = 283)	Any local complication (n = 4993)	BII (n = 58)	Any local complication (n = 1817)
Age at revision, y				
<30	27 (9.5)	204 (4.1)	0	7 (0.4)
30-39	77 (27.2)	873 (17.5)	3 (5.2)	113 (6.2)
40-49	74 (26.2)	1207 (24.2)	10 (17.2)	273 (15.0)
50-59	64 (22.6)	1392 (27.9)	21 (36.2)	617 (34.0)
≥60	41 (14.5)	1317 (26.4)	24 (41.4)	807 (44.4)
American Society of Anesthesiologists classification at revision				
I	203 (71.7)	3155 (63.2)	18 (31.0)	748 (41.2)
II	61 (21.6)	1564 (31.3)	34 (58.6)	910 (50.1)
III	11 (3.9)	222 (4.5)	6 (10.3)	141 (7.8)
IV-V	0	10 (0.2)	0	5 (0.3)
Unknown	8 (2.8)	42 (0.8)	0	13 (0.7)
Bilateral revision	282 (99.7)	4664 (93.4)	30 (51.7)	633 (34.8)
Received radiotherapy before revision				
No	268 (94.7)	4857 (97.3)	49 (84.5)	1323 (72.8)
Yes	14 (5.0)	51 (1.0)	4 (6.9)	290 (16.0)
Unknown	1 (0.4)	85 (1.7)	5 (8.6)	204 (11.2)

Abbreviation: BII, breast implant illness.

^a^ Women with unknown complications are not included.

### Prospective Cohort

A total of 47 564 cosmetic breast implants (24 120 women; mean [SD] age at implantation, 32.3 [9.7] years) and 5928 reconstructive breast implants (4688 women; mean age [SD] at implantation, 50.9 [11.5] years; data not shown) were included in the prospective cohort (Table 4). Most revisions were performed in the reconstruction group; 697 breast implants (11.8%) were revised after a median (IQR) time to reoperation of 1.1 (0.5-1.9) years. In the cosmetic group, 739 breast implants (1.6%) were revised with a median (IQR) time to reoperation of 1.8 (0.9-3.1) years. In the cosmetic group, the most common indication for revision was dissatisfaction with volume (289 revisions [39.1%]), followed by capsular contracture (148 revisions [20.0%]). In the reconstruction group, the 2 most frequent indications for revision were asymmetry (152 revisions [21.8%]) and capsular contracture (148 revisions [21.2%]). Only 35 cosmetic revisions (4.7%) and 5 reconstruction revisions (0.7%) were because of BII, corresponding to 0.1% of all inserted implants, respectively.

**Table 4.** Revision and Complication Rates in the Prospective Cohort

Revisions	Participants, No. (%)	
	Cosmetic augmentation (n = 47 564)	Breast reconstruction (n = 5928)
Revision rate	739 (1.6)	697 (11.8)
Time to reoperation, median (IQR), y	1.8 (0.9-3.1)	1.1 (0.5-1.9)
Complications as indication for revision^a^		
Dissatisfaction with volume	289 (39.1)	116 (16.6)
Capsular contracture	148 (20.0)	148 (21.2)
Baker grade IV	59 (8.0)	77 (11.1)
Asymmetry	91 (12.3)	152 (21.8)
Breast pain	66 (8.9)	132 (18.9)
Implant malposition	110 (14.9)	79 (11.3)
Deep wound infection	20 (2.7)	86 (12.3)
Skin necrosis	2 (0.3)	71 (10.2)
Additional complications		
Seroma	9 (1.2)	38 (5.5)
Skin scarring	5 (0.7)	35 (5.0)
Flap problem	0	26 (3.7)
Implant rupture	22 (3.0)	13 (1.9)
Breast implant illness		
Total^b^	35 (4.7)	5 (0.7)
With implant rupture	1 (0.1)	0
Hematoma	1 (0.1)	10 (1.4)
Silicone extravasation	8 (1.1)	6 (0.9)
BIA-ALCL suspected	8 (1.1)	1 (0.1)
BIA-ALCL confirmed	0	0
Complication unknown	159 (21.5)	97 (13.9)

Abbreviation: BIA-ALCL, breast implant–associated anaplastic large cell lymphoma.

^a^ Multiple complications can be registered per breast implant; consequently, percentages do not add up to 100%.

^b^ This includes observations of breast implant illness in combination with other complications.

In both the cosmetic and reconstruction groups, most revisions for BII were explantation (18 revisions [51.4%] and 4 revisions [80.0%], respectively) (Table 5). In the cosmetic group, 13 revisions because of BII (37.1%) were explantation with additional fat grafting. For breasts with local complications, the breast implant was most frequently replaced without additional techniques in both groups (421 revisions [77.2%] and 345 revisions [58.0%], respectively).

**Table 5.** Characteristics of Revisions in the Prospective Cohort, by BII and Local Complications as Indication for Revision^a^

Revision surgery type	Revisions, No. (%)			
	Cosmetic augmentation		Breast reconstruction	
	BII (n = 35)	Local complication (n = 545)	BII (n = 5)	Local complication (n = 595)
Replacement surgery				
With implant	0	421 (77.2)	0	345 (58.0)
With implant and mastopexy	0	47 (8.6)	0	2 (0.3)
With implant and fat grafting	0	2 (0.4)	0	29 (4.9)
With implant and autologous flap	0	2 (0.4)	1 (20.0)	33 (5.5)
With implant and combination of techniques	0	0	0	6 (1.0)
Explantation				
Without additional surgical techniques	18 (51.4)	60 (11.0)	4 (80.0)	164 (27.6)
With mastopexy	4 (11.4)	8 (1.5)	0	2 (0.3)
With fat grafting	13 (37.1)	3 (0.6)	0	1 (0.2)
With autologous flap	0	0	0	12 (2.0)
With combination of techniques	0	2 (0.4)	0	1 (0.2)

Abbreviation: BII, breast implant illness.

^a^ Revised breasts with unknown complications are not included.

## Discussion

In this cohort study, BII was registered as the reason for revision in 4.2% of cosmetic breast implant revisions in the legacy cohort of revised breasts (2015-2020) and in 4.7% of cosmetic breast implant revisions in the prospective cohort of women who underwent breast implantation between 2015 and 2019. Among reconstructive breast implants, 2.7% and 0.7% of revisions were performed because of BII in the legacy cohort and prospective cohort, respectively. The overall revision rate in the prospective cohort was 1.6% for cosmetic breast implants and 11.8% for reconstructive breast implants with a median time to reoperation of 1.8 years and 1.1 years, respectively. With this study, we provided descriptive data that can enable a clearer case definition that could reduce uncertainty about the BII diagnosis.

The current study showed that 4.7% of cosmetic breast implant revisions were performed because of BII, corresponding to 0.1% of the inserted implants. Previously reported prevalence rates of BII-like symptoms include postoperative rheumatic symptoms in 37.4% of cases,^21^ the development of a pattern of systemic complaints in more than 65% cases,^22^ and 3 or more BII symptoms in 38.5% to 84.7% of women with breast implants.^23^ The presence of selection bias in these previous studies, as well as discrepancies in outcome measures, follow-up time, and the definition used for BII, may explain the difference in BII prevalence rates between the current study and prior research. In addition, the number of women with BII we found according to breast implant revision surgery data are most likely an underestimation of the overall number of women with BII symptoms in our study population. Revision surgery is an invasive procedure with possible postoperative complications, and, therefore, not all women with BII symptoms may find this an acceptable solution. In addition, implant removal may have negative aesthetic consequences with possible body image issues as a result,^24^ and symptom relief is not guaranteed.^11,22,25^ Furthermore, the decision to undergo revision may be subject to socioeconomic status. In the Netherlands, explantation is reimbursed by health insurance companies only in cases of medical necessity, with BII rarely categorized as such.^26^ Finally, it is likely that not all women experiencing BII symptoms are aware that their health complaints might be related to their breast implants.

In our study, BII was diagnosed on the basis of the plastic surgeon’s assessment of patient-reported symptoms, with BII symptoms generally defined as medically unexplained systemic symptoms. These are health complaints that can be seen in the general public as well. In the Netherlands, 2.5% of adults have persistent, severe, medically unexplained systemic symptoms, and these patients are more often female.^27^ This raises the question what the results would be if we compared women with breast implants with control groups of women without breast implants and corrected for the presence of symptoms before implantation.

In the studied legacy cohort, most women had received their implants before 2015, and those who had their implant replaced or explanted because of BII did so after a median implant in situ time of 12.0 years (cosmetic) and 9.0 years (reconstruction). This corresponds to the median 13-year period between implantation and BII diagnosis found in the study of Colaris et al.^11^ However, time to reoperation does not have to be proportional to the duration women have experienced BII symptoms. In previous studies, the average time from implantation until onset of BII symptoms was approximately 4.5 years.^11,21,22,23^ This proposed symptom-free period in the first years after implantation could possibly explain the low number of revisions performed because of BII in the prospective cohort, where median time to reoperation was 1.8 years and 1.1 years because of the short follow-up period of the cohort. Still, more research is needed to better understand the development of symptoms and time to surgery.

More than three-quarters of total BII revisions were performed in the 2 most recent years, 2019 and 2020. This may be explained by something else than simply an increase in women with BII symptoms; our findings may have been confounded by increasing media coverage of BII. In recent years, BII has received a lot of attention in the Netherlands, especially after a television report on the topic aired in 2019 that gained more than 1 million views.^28,29^ Google Trends findings on search popularity of “breast implant illness” in the US also showed an approximately 8-fold increase of Google searches in early 2019 compared with early 2018.^30^ This increased media exposure may improve awareness among women with breast implants and medical professionals about a possible association between breast implants and health complaints. However, because validated diagnostic criteria for BII are lacking, media can easily present its own interpretation of the symptoms, thereby potentially spreading incomplete or even false information. Furthermore, the underlying danger of such heightened media attention is the generation of a certain expectation of health complaints among women with breast implants, which may lead to a nocebo effect, where the expected symptoms become reality.^31,32^

In the cosmetic group of the legacy cohort, women with BII were significantly younger, had better preoperative health at revision, and more often had received radiotherapy before revision compared with women who underwent revision because of local complications. An explanation for age differences between the 2 subgroups may be found in the immune defense system. The immune system functions better at a younger age, and antibody responses to antigens are substantially stronger.^33^ This is demonstrated by the poor response to vaccination in the elderly.^34^ Furthermore, lower levels of estradiol in postmenopausal women are suggested to enhance immunosenescence effects.^35^ However, the cause of BII and specific role of the immune system in the development of BII are not clear yet.^36,37^ In addition, certain patient characteristics that are more common among individuals aged 40 and younger compared with older adults, such as smoking and somatoform disorders, may have influenced the observed age difference.^38,39^ To fully understand who the women are who develop BII, future research should use multiple sources of health care data to evaluate patient characteristics prospectively over a longer period of time.

### Limitations

Some limitations of our study should be considered when interpreting the results. First, not all physicians recognize the existence of BII. As a result, registration of BII in DBIR is subject to the plastic surgeon’s individual knowledge of and beliefs in the disease. This may have led to an underestimation or overestimation of women who underwent revision because of BII. In addition, because of the generally poorer health of patients with breast cancer, plastic surgeons might be less likely to link health problems directly to the implants in reconstructive cases compared with women with a cosmetic indication. This could possibly explain why BII was more often registered in women with cosmetic implants vs women with reconstructive implants.

Second, on the basis of the data available in our study for body mass index and smoking (available from 2017), we were unable to fully examine the characteristics of women with BII and, therefore, could not determine risk factors related to BII. Phenotyping women with BII could help with understanding its cause and shift perceptions on who is considered a good candidate for breast implants. Research into the possible influence of lifestyle indicators, as well as comorbidities and demographic factors, on the development of BII is, therefore, urgently needed. Furthermore, information on implant characteristics was incomplete and for that reason, like body mass index and smoking status, not included in the analysis of this study. More women with breast implants will be registered as DBIR ages, including more patients with BII. Better registration of patient and implant characteristics is gathered in this expanding population.

Third, although we were able to calculate the time to reoperation for each complication in the legacy cohort, we did not have information on the duration of latency periods between implantation and the onset of complications. In addition, the prospective cohort’s follow-up period may have been too limited to determine the prevalence of BII as indication for revision surgery. We will continue to prospectively collect surgery data from this cohort.

## Conclusions

In this cohort study of women with silicone breast implants, despite increasing public interest in BII, it was an uncommon indication for revision compared with local complications, in both the short and long term. With this study, we present a first estimate of the magnitude of BII as a postoperative complication of breast implant surgery. The provided preliminary associative data could ultimately be used to estimate which implant recipients are most likely to develop BII. Despite the low risk of BII, the main concern is who BII actually affects. Large cohort studies are strongly recommended to investigate the prevalence of BII symptoms before and after implantation together with risk factors related to BII.

## Notes

Supplement. **eFigure 1.** Description of the Two Cohorts **eFigure 2.** Reasons of Missing Implantation Record in the Legacy Cohort
