# Supplementary material for: Prevalence of Local Postoperative Complications and Breast Implant Illness in Women With Breast Implants
Source: JAMA Netw Open. 2022 Oct 7;5(10):e2236519. doi: 10.1001/jamanetworkopen.2022.36519 (PMC9547313; doi:10.1001/jamanetworkopen.2022.36519)
Supplement: Supplement. — eFigure 1. Description of the Two Cohorts eFigure 2. Reasons of Missing Implantation Record in the Legacy Cohort [file jamanetwopen-e2236519-s001.pdf]

Supplemental Online Content

Lieffering AS, Hommes JE, Ramerman L, et al. Prevalence of local postoperative complications and breast implant illness in women with breast implants. *JAMA Netw Open*. 2022;5(10):e2236519. doi:10.1001/jamanetworkopen.2022.36519

eFigure 1. Description of the Two Cohorts

eFigure 2. Reasons of Missing Implantation Record in the Legacy Cohort

This supplemental material has been provided by the authors to give readers additional information about their work.

© 2022 Lieffering AS et al. *JAMA Network Open.*

eFigure 1. Description of the Two Cohorts

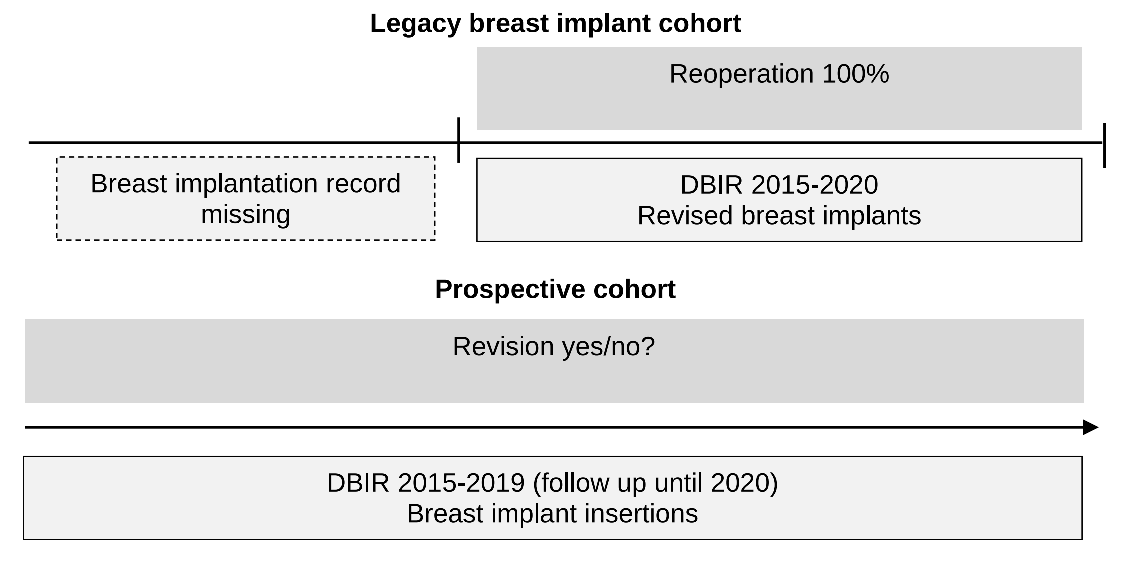

eFigure 2. Reasons of Missing Implantation Record in the Legacy Cohort

Total legacy cohort n = 15,827	
Implantation performed before start of DBIR	n = 10,004 (63.2%)
Implantation performed abroad	n = 1,220 (7.7%)
Clinic or surgeon did not register in DBIR	n = 1,754 (11.1%)
Reason unknown	n = 2,849 (18.0%)

© 2022 Lieffering AS et al. *JAMA Network Open.*
